# Association Between Endothelial Activation and Stress Index and 28-Day Mortality in Septic ICU patients: a Retrospective Cohort Study

**DOI:** 10.7150/ijms.85870

**Published:** 2023-07-31

**Authors:** Hong-Bo Xu, Yuan Ye, Fang Xue, Jinglan Wu, Zhijun Suo, Haigang Zhang

**Affiliations:** 1 Department of Critical Care Medicine, Huazhong University of Science and Technology Union Shenzhen Hospital, Shenzhen 518033, China.; 2 Department of Critical Care Medicine, The 6th Affiliated Hospital of Shenzhen University Health Science Center, Shenzhen 518033, China.

**Keywords:** Endothelial Activation and Stress Index, sepsis, endothelium, mortality, MIMIC-IV

## Abstract

**Background:** Endothelial Activation and Stress Index (EASIX) is a reliable alternative biomarker of endothelial dysfunction. Because endothelial activation is involved in sepsis pathophysiology, we aimed to investigate the association between EASIX and prognosis in septic patients.

**Methods:** Data were extracted from the Medical Information Mart for Intensive Care (MIMIC) IV database. EASIX scores were calculated using the formula: lactate dehydrogenase (U/L) × creatinine (mg/dL)/platelet count (10^9^/L). Patients were grouped into tertiles according to log2 transformed EASIX. The primary and secondary outcomes were 28-day and 90-day mortality. Cox proportional hazards models, Kaplan-Meier curves, restricted cubic spline curves, and subgroup analyses were conducted to evaluate the association between EASIX and prognosis in septic patients.

**Results:** A total of 7504 patients were included. Multivariable Cox proportional hazards analyses showed that higher log2-EASIX was associated with increased risk of 28-day mortality (HR, 1.10; 95% CI, 1.07-1.13; *P* < 0.001). Compared with tertile 1, the tertile 2 and 3 groups had higher risk of 28-day mortality [HR (95% CI) 1.24 (1.09-1.41); HR (95% CI) 1.51 (1.31-1.74)]; *P* for trend < 0.001). Similar results were found for 90-day mortality. Kaplan-Meier curves showed that patients with higher EASIX had lower 28-day and 90-day survival rates. A linear relationship was found between log2-EASIX and 28-day and 90-day mortality.

**Conclusion:** High EASIX was significantly associated with an increased risk of 28-day and 90-day all-cause mortality in patients with sepsis.

## Introduction

Sepsis, defined as a life-threatening organ dysfunction caused by a dysregulated host response to infection 1, is a common condition that results in significant morbidity and mortality globally. A recent report showed that there were a staggering 48.9 million sepsis cases and 11 million sepsis-related deaths worldwide, which accounted for almost 20% of all global deaths 2. Sepsis has become a major global health problem. Research suggested that swift and appropriate management of sepsis in its early stages could improve outcomes 1. Therefore, biomarkers that can rapidly identify septic patients with poor prognosis may be of great importance. It would facilitate more effective clinical decision-making and resource allocation, thereby slowing the progression of sepsis and enhancing patient outcomes. While several scoring systems have been shown to be associated with outcomes in sepsis, the use of them is inconvenient due to the integration of numerous parameters. Additionally, these scores exhibit suboptimal performance for the early and rapid detection of sepsis 3. For the convenience of clinical practice, it remains necessary and worthwhile to evaluate promising and convenient biomarkers for sepsis prognosis.

Recently, the endothelial activation and stress index (EASIX) score was suggested to be a novel biomarker of endothelial damage 4. It is calculated based on the following formula: serum lactate dehydrogenase (LDH) level (U/L) × creatinine level (mg/dL)/platelet count (10^9^/L), all of which are routine laboratory parameters 4. Endothelial dysfunction is involved in the pathophysiology of sepsis 5-7. Sepsis-associated endothelial changes may be adaptive in the early stages, but they can become excessive, ultimately leading to impaired microcirculatory blood flow, tissue hypoperfusion and life-threatening organ failure 8. Therefore, biomarkers associated with endothelial activation may be valuable in the early identification of septic patients with poor prognosis. In addition, the three components of EASIX have been suggested as prognostic factors in sepsis 9-15, and the EASIX score has been shown to predict the development of sepsis after allogeneic stem cell transplantation 16. Against this background, our study aimed to investigate the association between EASIX and 28-day and 90-day all-cause mortality in septic patients. We hypothesized that elevated EASIX is related to increased risk of mortality in patients with sepsis.

## Methods

### Data source

Research data were extracted from the Multiparameter Intelligent Monitoring in Intensive Care (MIMIC) IV database (version 2.1). MIMIC IV is a contemporary electronic health record dataset containing deidentified clinical data of patients admitted to the intensive care unit (ICU) of Beth Israel Deaconess Medical Center between 2008 and 2019 17. According to the guidelines for using the MIMIC database, one author (Xu HB) completed a human subjects research training course (record ID 35959043) and became a credentialed user of PhysioNet. After signing the Data Use Agreement (DUA), we were qualified to use the MIMIC-IV database. Use of the dataset was approved by the Institutional Review Boards of Beth Israel Deaconess Medical Center (Boston, MA). Informed consent was not required because patient information in this database was de-identified. This study was reported according to the Strengthening the Reporting of Observational Studies in Epidemiology guidelines 18.

### Study population

Adult patients with sepsis were included in our study. The diagnosis of sepsis was made according to Sepsis 3.0 criteria 1. Exclusion criteria were as follows: (a) repeated ICU admission; (b) with missing serum creatinine, LDH or platelet data during the first 24 hours after ICU admission; (c) age < 18 years; (d) length of ICU stay < 24 hours.

### Variables extraction

PostgreSQL(version 9.6) was used to extract the following variables from the MIMIC IV database, including age, gender, ethnicity, temperature, heart rate, respiratory rate, mean arterial pressure, pulse oxygen saturation (SpO2), laboratory results (white blood cell, red blood cell distribution width, platelet, hemoglobin, sodium, potassium, chloride, calcium, albumin, serum creatinine, blood urea nitrogen, glucose, prothrombin time, partial thromboplastin time, LDH), Simplified Acute Physiology Score II (SAPS II), Sequential Organ Failure Assessment (SOFA) Score, renal replacement therapy (RRT) use, ventilator use, co-morbidities (diabetes, congestive heart failure, hypertension, chronic pulmonary disease, liver disease, renal failure, malignant cancer, septic shock). If a variable was measured multiple times within 24 h after ICU admission, only the first value was extracted. Comorbidities, except septic shock, were diagnosed according to International Classification of Diseases (ICD)-9 and ICD-10 codes. Septic shock is a subset of sepsis and was defined in the present study as receipt of any vasopressor or inotropic infusion, including norepinephrine, epinephrine, dobutamine, phenylephrine, vasopressin, dopamine and milrinone, within 24 hours after ICU admission 19, 20. EASIX score was calculated using the formula “LDH [U/L] × creatinine [mg/dl]/platelet count [10^9^/L]”.

### Outcomes

The primary outcome was 28-day all-cause mortality after ICU admission. The secondary outcome was 90-day all-cause mortality after ICU admission.

### Management of missing data and outliers

Details of missing values are provided in supplementary materials Table S1. In order to reduce bias, variables with missing values over 20% were excluded from the study. Variables with missing values less than 10% were replaced by the mean or median, if appropriate. The remaining indicators (10-20% missing) were imputed using the multiple imputation method. Additionally, variables with abnormal values were adjusted by the winsor2 command with replace cuts (1,99). The STATA software (version 14) was used to handle missing and abnormal data.

### Statistical analysis

Continuous variables were shown as mean ± standard deviation (SD) or median (interquartile range), while categorical data were expressed as numbers (%). AS EASIX had a skewed distribution, it was log2 transformed before analysis 4, 21. All enrolled subjects were divided into three groups by the tertiles of log2-EASIX. Comparisons between the three groups were performed using one-way analysis of variance test or Kruskal-Wallis test for continuous variables and χ2 or Fisher exact test for categorical variables, as appropriate. The 28-day and 90-day survival curves were estimated by the Kaplan-Meier method for the three tertile groups and compared using the log-rank test. The association between log2-EASIX and 28-day and 90-day mortality was investigated using Cox proportional hazards models. The assumption of multicollinearity was assessed by estimating the variance inflation factor (VIF), and VIF value above 5 was regarded as an indicator of multicollinearity. Both unadjusted and multivariable adjusted models were performed to evaluate the robustness of the results. Adjusted covariates were selected on the basis of clinical relevance and a change in the effective estimate of at least 10%. In model 1, we adjusted for age, gender and ethnicity. In model 2, we further adjusted for hypertension, congestive heart failure, chronic pulmonary disease, diabetes, liver disease, renal failure, malignant cancer and septic shock. In model 3, we additionally adjusted for ventilator use, RRT use, SOFA and SAPS II. In model 4, we further adjusted for temperature, white blood cell, red blood cell distribution width, blood urea nitrogen, mean arterial pressure, and hemoglobin. The likely non-linear relationship between log2-EASIX and survival outcome was explored by restricted cubic spline analysis. A two-piecewise linear regression model would be conducted to determine the threshold effect of log2-EASIX on sepsis prognosis if a non-linear correlation was observed. Subgroup analyses were also conducted to assess the robustness of our findings, including age, gender, ethnicity, hypertension, congestive heart failure, chronic pulmonary disease, diabetes, liver disease, renal failure, malignant cancer, septic shock, SAPS II, SOFA, ventilator use, RRT use. Data analyses were performed using R 4.2.1 (http://www.R-project.org, The R Foundation) and Free Statistics software (version 1.7.1). A two-sided *P* values less than 0.05 was considered as statistically significant.

## Results

### Baseline characteristics of the enrolled patients

**Figure 1:**
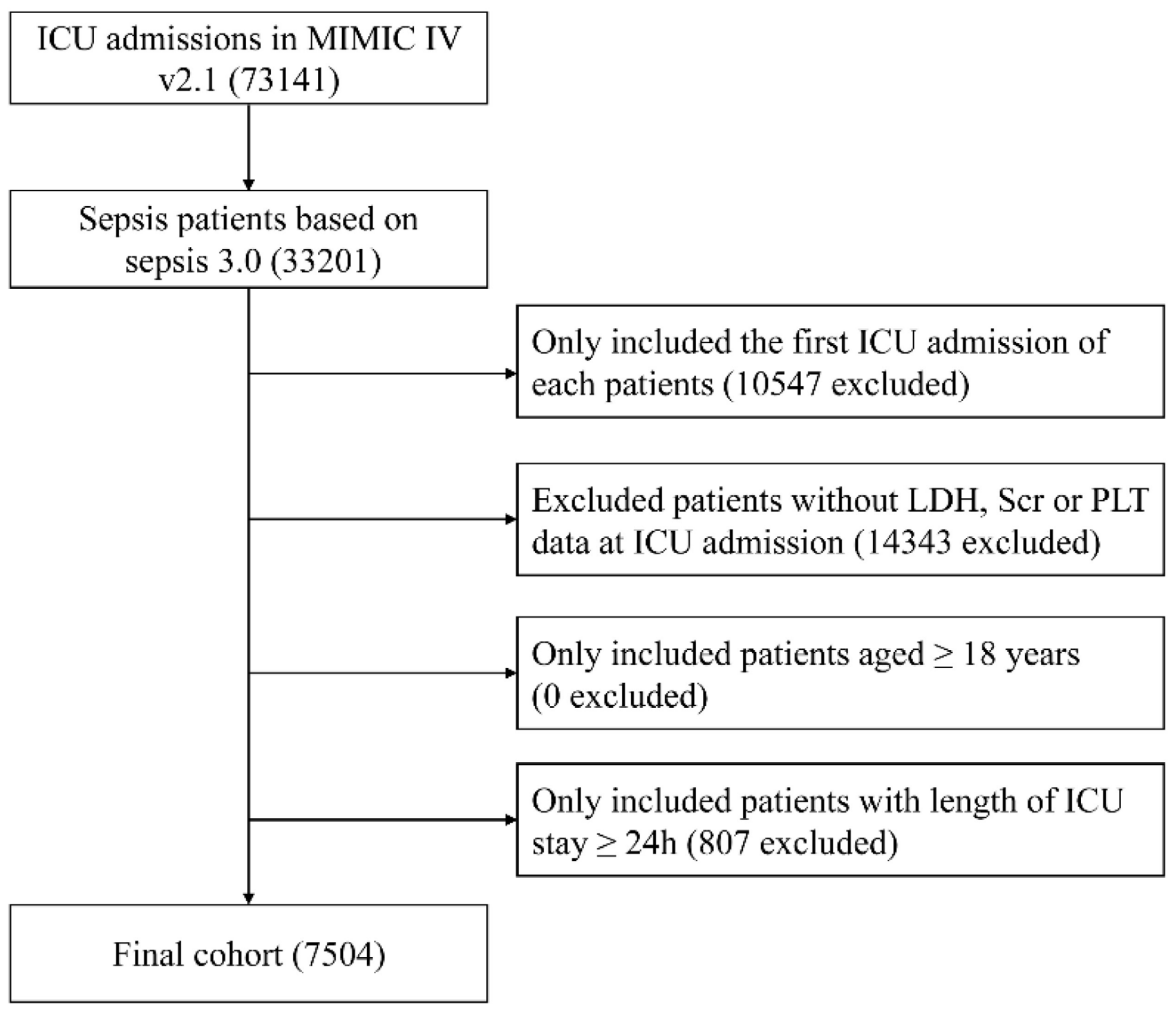
Flow diagram of subject selection. MIMIC, Medical Information Mart for Intensive Care; ICU, intensive care unit; LDH, lactate dehydrogenase; SCr, serum creatinine; PLT, platelet.

A total of 7540 septic patients were included in the final cohort according to the aforementioned criteria (Figure 1). The baseline characteristics of the enrolled patients are presented in Table 1. In general, the mean age of the enrolled patients was 64.5 ± 17.1 years, approximately 57.0% of the patients were male and 62.8% of the patients were white. Patients with higher log2-EASIX had higher SAPS II score, SOFA score, increased use of ventilator and RRT. Among those with higher log2-EASIX, there was a relatively higher prevalence of hypertension, congestive heart failure, diabetes, liver disease, renal failure and malignant cancer. Additionally, patients with higher log2-EASIX were more likely to be complicated with shock. As log2-EASIX increased, red cell distribution width, glucose, blood urea nitrogen, prothrombin time, partial thromboplastin time, LDH and serum creatinine levels increased, while platelet levels decreased. For the outcomes of the present study, the 28-day and 90-day mortality were 25.9% and 33.7%, respectively. Patients with elevated log2-EASIX had a higher 28-day/90-day mortality (all *p* < 0.001).

### Association between EASIX and survival outcome

**Figure 2:**
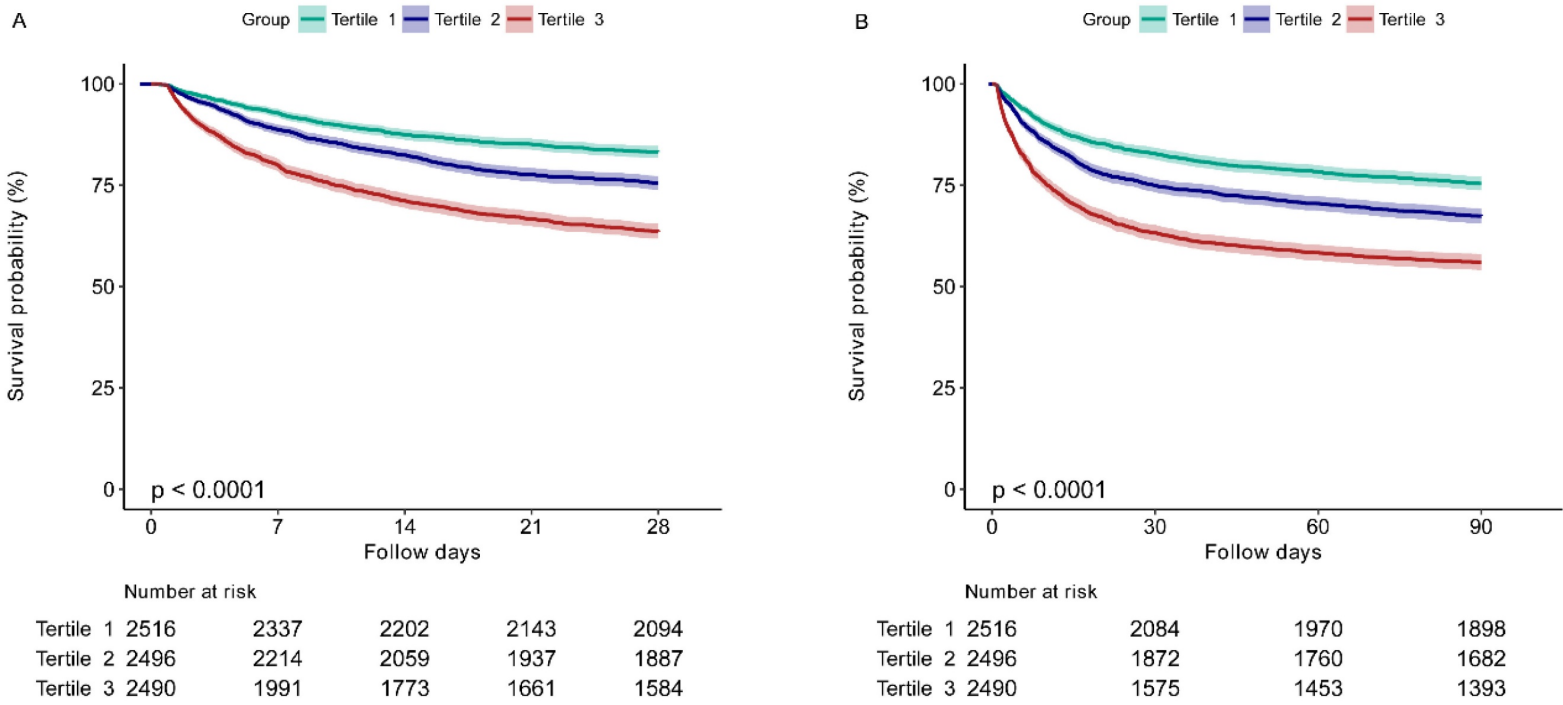
The 28-day (A) and 90-day (B) survival curves of sepsis patients stratified by tertiles of EASIX. EASIX, Endothelial Activation and Stress Index. EASIX index: Tertile 1 (<1.42), Tertile 2 (1.42-4.08), Tertile 3 (≥4.08).

The Kaplan-Meier survival curves for 28-day and 90-day mortality according to the EASIX tertiles are presented in Figure 2. It was showed that patients in the higher EASIX group had a significant lower 28-day survival rate (*P* for log-rank test < 0.0001) (Figure 2A). Similar results were found for the 90-day survival curves (*P* for log-rank test < 0.0001) (Figure 2B).

Several Cox proportional hazards regression models were performed to assess the independent effect of EASIX on 28-day and 90-day mortality in patients with sepsis (Table 2). It was shown that log2-EASIX was positively related to the risk of 28-day mortality. Compared with the first tertile group, the unadjusted hazard ratio (HR) [95% confidence interval (CI)] were 1.52 (95% CI 1.34, 1.72) and 2.50 (95%CI 2.23, 2.81) in the second and third tertile groups of log2-EASIX, respectively. This significant association remained even after controlling for variables, such as age, gender, ethnicity, hypertension, congestive heart failure, chronic pulmonary disease, diabetes, liver disease, renal failure, malignant cancer, septic shock, ventilator use, RRT use, SOFA score, SAPS II score, temperature, white blood cell count, red cell distribution width, blood urea nitrogen, mean arterial pressure, and hemoglobin. In model 4, the adjusted HRs (95% CI) were 1.24 (95% CI 1.09, 1.41) and 1.51 (95% CI 1.31, 1.74) for tertile 2 and tertile 3, respectively, with tertile 1 as the reference group. Furthermore, when analyzed as a continuous variable, log2-EASIX was also associated with 28-day mortality in both unadjusted and adjusted models. The HRs (95% CI) in the five models were 1.21 (1.19, 1.23), 1.23 (1.21, 1.26), 1.20 (1.18, 1.23), 1.09 (1.06, 1.12), and 1.10 (1.07, 1.13), respectively. Similar results were observed when evaluating the association between EASIX and 90-day mortality by Cox proportional hazards regression (Table 2).

Restricted cubic spline analysis for the Cox model was performed to explore the shape of the relationship between log2-EASIX and survival outcome. It was found that the 28-day mortality risk increased linearly with increasing log2-EASIX Figure S1. Additionally, a similar linear association was also observed between log2-EASIX and the risk of 90-day mortality Figure S2.

### Subgroup analyses

**Figure 3:**
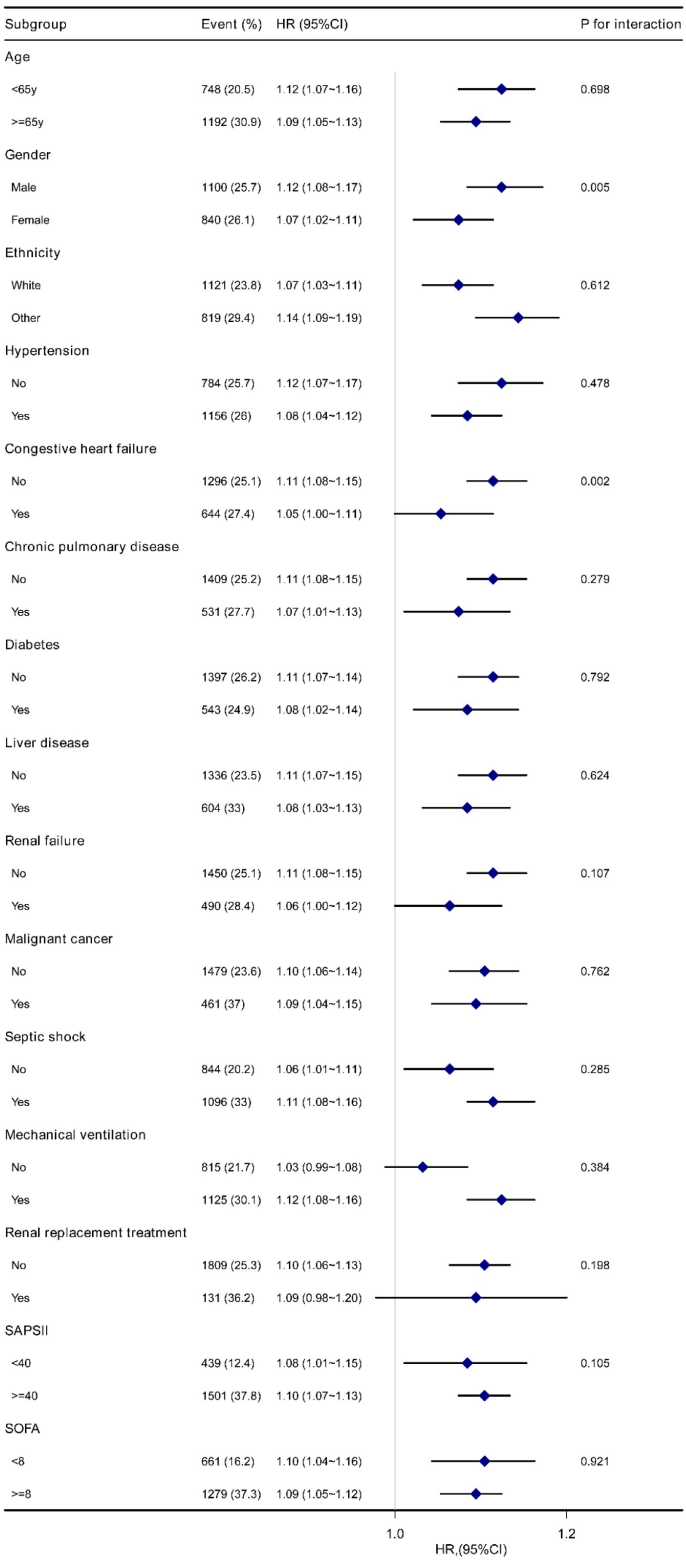
Forest plot for subgroup analysis of the association between Log2-EASIX and 28-day mortality in sepsis patients. EASIX, Endothelial Activation and Stress Index; HR, hazard ratio; CI, confidence interval; SAPS II, Simplified Acute Physiology Score II; SOFA, Sequential Organ Failure Assessment.

The stability of the association between log2-EASIX and 28-day mortality was evaluated by subgroup analyses. The trend of the effect size was consistent in all subgroups (Figure 3). Significant interactions were observed among gender (*P*=0.005) and congestive heart failure (*P*=0.002). Male patients with sepsis had a relatively higher risk of 28-day mortality [HR (95% CI) male 1.12 (1.08-1.17) vs. female 1.07 (1.02-1.11)]. A similar trend was observed in patients without congestive heart failure [HR (95% CI) without congestive heart failure 1.11 (1.08~1.15) vs. with congestive heart failure 1.05 (1.00~1.11)]. No significant interactions were found in other subgroups (*P* for interaction > 0.05). Similar results were found for 90-day mortality Figure S3.

## Discussion

To the best of our knowledge, the present study is the first to investigate the association between EASIX and prognosis in septic patient. Our results showed that a higher EASIX score was independently associated with an increased risk of 28-day and 90-day all-cause mortality. Furthermore, the results were consistent across different subgroups.

The endothelium plays a key role in sepsis. Sepsis-induced endothelial activation leads to changes in inflammation, vascular permeability, hemostatic balance, leukocyte trafficking and microcirculatory flow. It has been suggested that sepsis is a dysfunctional endothelial response to harmful microorganisms 16. Therefore, there is a compelling biological rationale for focusing on markers of endothelial dysfunction as biomarkers of sepsis. Zhou and colleagues reported that serum soluble thrombomodulin(sTM) and syndecan-1 could be potential biomarkers for the early diagnosis of sepsis 22. In another study, endocan was found to be a valuable predictor of sepsis severity and mortality 23. Recently, serum levels of 11 biomarkers associated with endothelial dysfunction were assessed and used to identify septic patients with distinct endothelial response profiles. Three different profiles were identified and showed the ability to discriminate septic patient with high risk of mortality 24. Other relevant studies can be found in an excellent review by Xing et al. 25. However, measuring endothelial dysfunction is a major challenge as there is no routine blood test available for this purpose.

Recently, an index named EASIX was introduced by Luft et al. as a novel marker of systemic endothelial activation in the setting of allogeneic hematopoietic stem cell transplantation 4. EASIX is calculated using serum LDH, creatinine and platelet levels, all of which are readily available in routine clinical practice. These parameters were part of the classical diagnostic criteria of thrombotic microangiopathy and were considered as the most interesting parameters associated with endothelial pathology 4, 26, 27. Increased serum levels of LDH during endothelial damage can be attributed to direct release from both endothelial cells and circulating cells, such as thrombocytes and leukocytes 28, 29. High level of creatinine serves as a link between endothelial pathology and renal function, as endothelial dysfunction acts an important role in conditions such as acute kidney injury 30, diabetic nephropathy 31, chronic kidney disease 32, and transplant glomerulonephropathy 33. Low platelet counts may be partly due to endothelial damage and complement activation in many diseases, such as thrombotic microangiopathies 29, chronic graft-versus-host disease 34, and acute respiratory distress syndrome 35. It should be recognized that there are alternative explanations for isolated changes in LAH, creatinine, and platelet count. This warning also applies to sepsis. However, EASIX is primarily a prognostic marker designed to be applicable in clinical practice with minimal costs. Furthermore, a high EASIX score was found to correlate with increased endothelial activation markers, such as suppressor of tumorigenicity-2, angiopoietin-2, soluble thrombomodulin, interleukin-18, chemokine-X-C-ligand 8 (CXCL8), insulin-like-growth-factor-1^36^, ^37^. This suggests that EASIX may be driven, at least in part, by endothelial activation. To date, EASIX has been confirmed as a simple and convenient prognostic index for survival in several diseases, including malignant hematologic diseases 2, 37-40, solid tumors 41, 42, and COVID-19 disease 21, 36, 43, 44. However, there are no reported studies focusing on the association between EASIX and prognosis in sepsis. In the present study, we found that high EASIX was associated with an increased risk of 28-day and 90-day all-cause mortality in septic patients.

The three components of EASIX have been evaluated as prognostic factors in sepsis in previous studies. It has been reported that admission LDH level can be used to assess the progress and prognosis of patients with severe sepsis 9, 10. Kidney injury, including acute kidney injury (AKI) and chronic kidney disease (CKD), has emerged as a frequent occurrence in septic patients and is associated with increased morbidity and mortality 45-47. Although serum creatinine levels may underestimate renal injury in sepsis 48, elevated serum creatinine has been shown to be a negative prognostic factor in septic patients 11, 12. In addition, thrombocytopenia was also reported to be an independent prognostic factor for septic patients 13-15. These findings suggests that measurements of the three components may have a synergistic role in predicting the prognosis of sepsis. Therefore, it is biologically feasible that EASIX is a reliable prognostic indicator in septic patients.

The stratified analysis showed the consistent trend of the effect size in all subgroups, indicating the robustness of the association between EASIX and prognosis in septic patients. A possible interaction was also found between EASIX and sex and congestive heart failure. It could be speculated that male septic patients or those without congestive heart failure may benefit more from EASIX scoring. To our knowledge, this is the first study to explore these interactions. The underlying mechanism is not unclear. Further studies are needed.

Our study has several limitations. Firstly, this is a single-center study, and potential selection bias cannot be avoided. Thus, some caution should be taken in generalizing these findings. Secondly, due to the nature of retrospective studies, residual confounding factors remained, although we made every effort to adjust for possible confounders. Thirdly, EASIX was evaluated upon ICU admission, whereas the dynamic changes were not further assessed during the patient's ICU stay; however, our goal was to investigate the potential of EASIX to discriminate septic patients with unfavorable prognosis in the early stage. Fourthly, the parameters required to calculate EASIX are not specific to endothelial damage. They can be influenced by a variety of pathophysiological processes, such as insufficient or replaced hematopoiesis, cell turnover, liver disease and kidney function. Thus, the likely confounding effects of underlying conditions on EASIX should be acknowledged. Despite these limitations, the contribution of our study to the development of appropriate markers from routine laboratory test for identifying septic patients at high risk of poor prognosis cannot be neglected. Further extensive multicenter studies are needed to confirm the role of EASIX as a prognostic marker for septic patients.

## Conclusion

High EASIX was significantly associated with an increased risk of 28-day and 90-day all-cause mortality in patients with sepsis. This finding supports a promising role for EASIX in early risk stratification and prognosis prediction in septic patients.

## Supplementary Material

Supplementary figures and table.

## Figures and Tables

**Table 1:** Characteristics of the included patients.

Variables	Total (n = 7504)	EASIX			*P* value
		<1.42 (n = 2517)	≥1.42, <4.08 (n = 2497)	≥4.08 (n = 2490)	
**Age, years**	64.5 ± 17.1	64.4 ± 17.6	65.8 ± 17.0	63.2 ± 16.5	< 0.001
**Gender, n (%)**					< 0.001
Female	3223 (43.0)	1270 (50.5)	985 (39.4)	968 (38.9)	
Male	4281 (57.0)	1247 (49.5)	1512 (60.6)	1522 (61.1)	
**Ethnicity, n (%)**					< 0.001
White	4715 (62.8)	1639 (65.1)	1591 (63.7)	1485 (59.6)	
Other	2789 (37.2)	878 (34.9)	906 (36.3)	1005 (40.4)	
**Vital sings**					
Temperature,℃	36.8 ± 0.9	36.8 ± 0.8	36.8 ± 0.9	36.8 ± 1.0	0.002
Heart rate, beats/min	94.2 ± 21.6	94.3 ± 22.4	93.1 ± 21.2	95.3 ± 21.2	0.002
Respiratory rate, beats/min	20.0 (16.0, 24.0)	20.0 (16.0, 24.0)	20.0 (16.0, 24.0)	21.0 (17.0, 25.0)	< 0.001
MAP, mmHg	81.9 ± 18.6	83.4 ± 18.1	82.0 ± 18.4	80.2 ± 19.1	< 0.001
SPO2	97.0 (95.0, 100.0)	98.0 (95.0, 100.0)	97.0 (95.0, 100.0)	97.0 (94.0, 100.0)	< 0.001
**Co-morbidities, n (%)**					
Hypertension	4448 (59.3)	1392 (55.3)	1543 (61.8)	1513 (60.8)	< 0.001
Congestive heart failure	2348 (31.3)	619 (24.6)	873 (35)	856 (34.4)	< 0.001
Chronic pulmonary disease	1915 (25.5)	686 (27.3)	627 (25.1)	602 (24.2)	0.037
Diabetes	2179 (29.0)	644 (25.6)	756 (30.3)	779 (31.3)	< 0.001
Liver disease	1828 (24.4)	310 (12.3)	594 (23.8)	924 (37.1)	< 0.001
Renal failure	1724 (23.0)	252 (10)	601 (24.1)	871 (35)	< 0.001
Malignant cancer	1247 (16.6)	386 (15.3)	366 (14.7)	495 (19.9)	< 0.001
Septic shock	3326 (44.3)	936 (37.2)	1109 (44.4)	1281 (51.4)	< 0.001
**Laboratory Results**					
WBC (k/ul)	11.7 (7.8, 16.9)	12.1 (8.5, 16.8)	11.8 (7.9, 16.9)	11.1 (7.0, 17.3)	< 0.001
RDW (%)	15.0 (13.8, 16.9)	14.5 (13.5, 16.1)	14.8 (13.7, 16.6)	15.7 (14.2, 17.8)	< 0.001
Hemoglobin (g/dL)	10.8 ± 2.5	11.1 ± 2.4	11.0 ± 2.6	10.3 ± 2.6	< 0.001
Sodium (mEq/L)	137.6 ± 5.8	137.8 ± 5.5	138.1 ± 5.7	137.0 ± 6.3	< 0.001
Potassium (mEq/L)	4.3 ± 0.9	4.2 ± 0.7	4.3 ± 0.9	4.6 ± 1.1	< 0.001
Chloride (mEq/L)	102.5 ± 7.3	102.7 ± 6.7	103.2 ± 7.2	101.5 ± 7.8	< 0.001
Calcium (mEq/L)	8.2 ± 0.9	8.3 ± 0.9	8.2 ± 0.9	8.1 ± 1.0	< 0.001
Glucose (mg/dL)	131.0 (104.0, 175.0)	128.0 (103.0, 165.0)	131.0 (106.0, 176.0)	133.0 (104.0, 185.0)	0.003
BUN (mg/dL)	24.0 (15.0, 42.0)	17.0 (12.0, 25.0)	24.0 (16.0, 38.0)	40.0 (24.0, 64.0)	< 0.001
PT (second)	14.6 (12.8, 18.2)	13.9 (12.3, 15.8)	14.6 (12.8, 17.8)	16.0 (13.5, 21.9)	< 0.001
PPT (second)	31.3 (27.3, 38.8)	30.3 (26.7, 34.7)	31.3 (27.0, 38.4)	33.6 (28.5, 44.3)	< 0.001
LDH (IU/L)	295.0 (214.0, 471.0)	221.0 (176.0, 281.0)	301.0 (229.0, 425.0)	500.0 (311.0, 997.0)	< 0.001
SCr (mg/dL)	1.2 (0.8, 2.0)	0.8 (0.7, 1.1)	1.2 (0.9, 1.7)	2.1 (1.3, 3.6)	< 0.001
PLT (k/ul)	187.0 (119.0, 268.0)	259.0 (196.0, 341.0)	177.0 (127.0, 241.0)	119.0 (63.2, 187.0)	< 0.001
log2-EASIX	1.5 ± 2.0	-0.4 ± 0.7	1.2 ± 0.4	3.7 ± 1.5	< 0.001
**Scores**					
SOFA	7.0 (4.0, 11.0)	5.0 (3.0, 7.0)	7.0 (5.0, 10.0)	10.0 (7.0, 14.0)	< 0.001
SAPSII	40.0 (32.0, 51.0)	36.0 (28.0, 45.0)	40.0 (31.0, 49.0)	47.0 (37.0, 59.0)	< 0.001
**Therapies, n (%)**					
Mechanical ventilation	3741 (49.9)	1213 (48.2)	1236 (49.5)	1292 (51.9)	0.03
Renal replacement therapy	362 (4.8)	7 (0.3)	37 (1.5)	318 (12.8)	< 0.001
**Outcomes**					
28day-mortality, n (%)	1940 (25.9)	423 (16.8)	610 (24.4)	907 (36.4)	< 0.001
90day-mortality, n (%)	2531 (33.7)	619 (24.6)	815 (32.6)	1097 (44.1)	< 0.001

Data are presented as the mean ± standard deviation (SD) for normal variables, median (IQR) for skewed variables, and numbers (proportions) for categorical variables. EASIX, Endothelial Activation and Stress Index; MAP, mean blood pressure; WBC, white blood cell; RDW, red cell distribution width; PLT, platelet; SCr, serum creatinine; BUN, blood urea nitrogen; PT, prothrombin time; PTT, partial thromboplastin time; LDH, lactate dehydrogenase; PLT, platelet; SAPS II, Simplified Acute Physiology Score II; SOFA, Sequential Organ Failure Assessment.

**Table 2:** Risk of 28-day and 90-day Mortality according to Log2-EASIX.

	Non-adjusted		Model 1		Model 2		Model 3		Model 4	
	HR (95% CI)	*P* value	HR (95% CI)	*P* value	HR (95% CI)	*P* value	HR (95% CI)	*P* value	HR (95% CI)	*P* value
**28-day mortality**										
Log2(EASIX)	1.21 (1.19~1.23)	<0.001	1.23 (1.21~1.26)	<0.001	1.20(1.18~1.23)	<0.001	1.09 (1.06~1.12)	<0.001	1.10 (1.07~1.13)	<0.001
Tertile of Log2-EASIX										
< 0.51	Reference		Reference		Reference		Reference		Reference	
0.51-2.03	1.52 (1.34~1.72)	<0.001	1.48 (1.31~1.68)	<0.001	1.46 (1.28~1.65)	<0.001	1.24 (1.09~1.41)	0.001	1.24 (1.09~1.41)	0.001
≥2.03	2.50 (2.23~2.81)	<0.001	2.55 (2.27~2.87)	<0.001	2.35 (2.08~2.66)	<0.001	1.50 (1.31~1.73)	<0.001	1.51 (1.31~1.74)	<0.001
*P* for trend		<0.001		<0.001		<0.001		<0.001		<0.001
**90-day mortality**										
Log2(EASIX)	1.17 (1.15~1.19)	<0.001	1.20 (1.18~1.22)	<0.001	1.16 (1.14~1.19)	<0.001	1.05 (1.03~1.08)	<0.001	1.06 (1.03~1.09)	<0.001
Tertile of Log2-EASIX										
< 0.51	Reference		Reference		Reference		Reference		Reference	
0.51-2.03	1.41 (1.27~1.56)	<0.001	1.37 (1.23~1.52)	<0.001	1.34 (1.2~1.49)	<0.001	1.14 (1.02~1.27)	0.018	1.15 (1.03~1.28)	0.013
≥2.03	2.14 (1.94~2.36)	<0.001	2.20 (1.99~2.43)	<0.001	2.00 (1.8~2.22)	<0.001	1.29 (1.15~1.45)	<0.001	1.29 (1.14~1.46)	<0.001
*P* for trend		<0.001		<0.001		<0.001		<0.001		<0.001

Model 1 adjusted for age, gender and ethnicity; Model 2 adjusted for model 1 plus hypertension, congestive heart failure, chronic pulmonary disease, diabetes, liver disease, renal failure, malignant cancer, and septic shock. Model 3 adjusted for model 2 plus Mechanical ventilation, Renal replacement therapy, SOFA and SAPSII score. Model 4 adjusted for model 3 plus temperature, WBC, RDW, BUN, MAP, and hemoglobin. HR, hazard ratio; CI, confidence interval; EASIX, Endothelial Activation and Stress Index; SOFA, Sequential Organ Failure Assessment; SAPS II, Simplified Acute Physiology Score II; WBC, white blood cell; RDW, red cell distribution width; BUN, blood urea nitrogen; MAP, mean arterial pressure.
