# Supplementary material for: Association Between Endothelial Activation and Stress Index and 28-Day Mortality in Septic ICU patients: a Retrospective Cohort Study
Source: Int J Med Sci. 2023 Jul 31;20(9):1165–73. doi: 10.7150/ijms.85870 (PMC10416722; doi:10.7150/ijms.85870)

Supplementary materials

Table S1. The proportion of missing values of the extracted variables

Figure S1. Association between Log2-EASIX and 28-day mortality in patients with sepsis. Data were fitted by a multivariable-adjusted restricted cubic spline Cox's regression. A linear association between Log2-EASIX and 28-day mortality was observed with a trend to rise. Log2-EASIX was entered as continuous variable, variables in model 4 of Table 2 were adjusted. The curves line and shaded ribbons around represented the estimated values and their corresponding 95% confidence intervals. EASIX, Endothelial Activation and Stress Index.

Figure S2. Association between Log2-EASIX and 90-day mortality in patients with sepsis. Data were fitted by a multivariable-adjusted restricted cubic spline Cox's regression. A linear association between Log2-EASIX and 90-day mortality was observed with a trend to rise. Log2-EASIX was entered as continuous variable, variables in model 4 of Table 2 were adjusted. The curves line and shaded ribbons around represented the estimated values and their corresponding 95% confidence intervals. EASIX, Endothelial Activation and Stress Index.

Figure S3. Forest plot for subgroup analysis of the association between Log2-EASIX and 90-day mortality in sepsis patients. EASIX, Endothelial Activation and Stress Index; HR, hazard ratio; CI, confidence interval; SAPS II, Simplified Acute Physiology Score II; SOFA, Sequential Organ Failure Assessment.

Table S1 The proportion of missing values of the extracted variables

	Missing data (n)	Missing data (%)
Age (years)	0	0
Gender	0	0
Ethnicity	0	0
Temperature (℃)	54	0.7
Heart rate, beats/min	2	<0.1
Respiratory rate, beats/min	16	0.2
MAP (mmHg)	17	0.2
Spo2 (%)	3	<0.1
Hypertension	0	0
Congenstive heart failure	0	0
chronic pulmonary disease	0	0
Diabetes	0	0
Liver disease	0	0
Renal failure	0	0
Malignant cancer	0	0
Septic shock	0	0
WBC (k/ul)	2	<0.1
RDW (%)	5	<0.1
Hemoglobin (g/dL)	5	<0.1
Sodium (mEq/L)	2	<0.1
Potassium (mEq/L)	2	<0.1
Chloride (mEq/L)	0	0
Calcium (mg/dL)	109	1.5
Glucose (mg/dL)	1	<0.1
Albumin(g/dL)	1724	23%
BUN (mg/dL)	1	<0.1
PT (second)	225	3
PTT (second)	252	3.3
PLT (k/ul)	0	0
SCr (mg/dL)	0	0
LDH (IU/L)	0	0
SOFA	0	0
SAPS II	0	0
Mechanical ventilation	0	0
renal replacement treatment	0	0

Figure S1

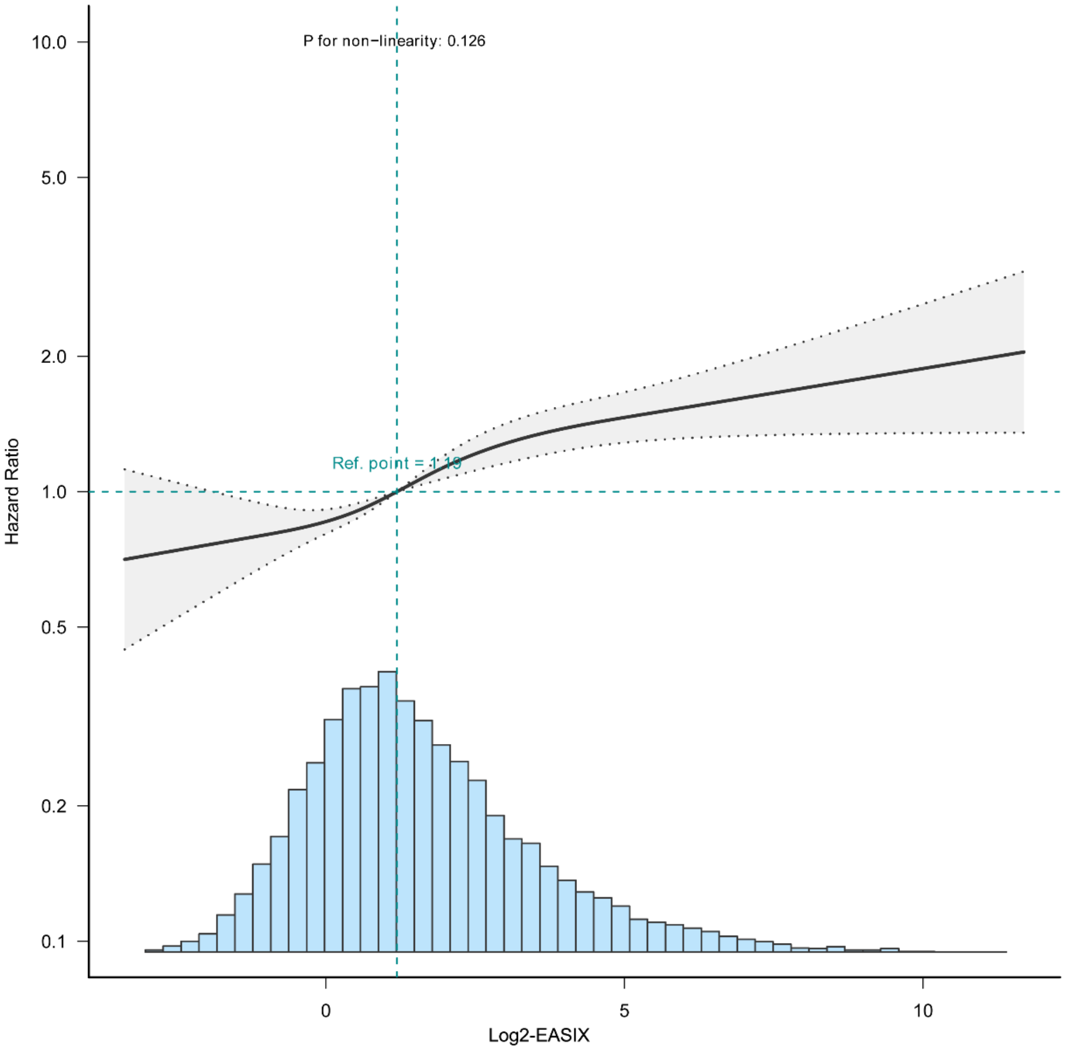

P for non−linearity: 0.126
Ref. point = 1.19
Hazard Ratio
10.0
5.0
2.0
1.0
0.5
0.2
0.1
0
5
10
Log2-EASIX

Figure S2

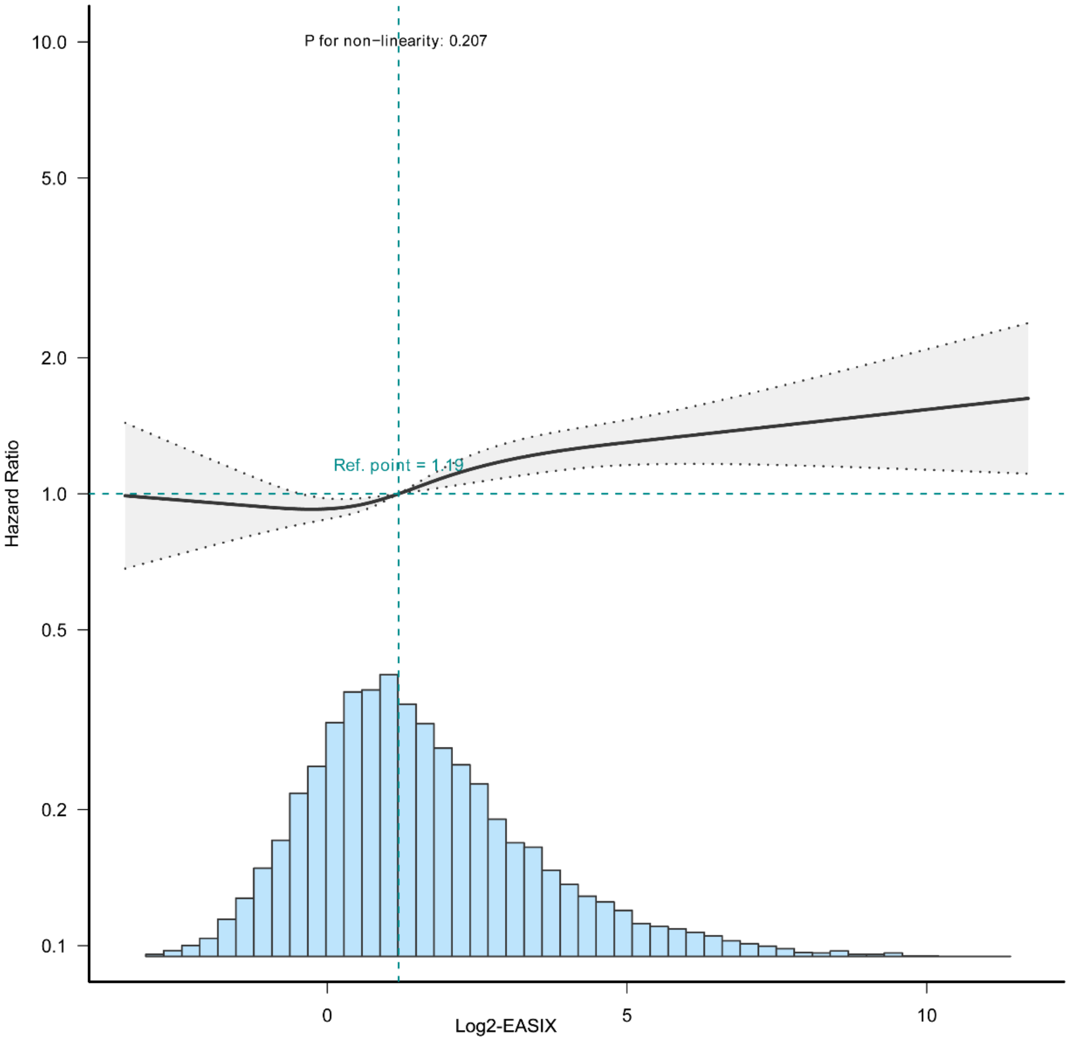

P for non−linearity: 0.207
Ref. point = 1.19
Hazard Ratio
10.0
5.0
2.0
1.0
0.5
0.2
0.1
0
5
10
Log2-EASIX

Figure S3

Subgroup	Total	Event (%)	HR (95%CI)	P for interaction
Age				
<65y	3643	963 (26.4)	1.07 (1.03~1.11)	0.909
>=65y	3861	1568 (40.6)	1.06 (1.02~1.09)	
Gender				
Male	4281	1428 (33.4)	1.07 (1.03~1.11)	0.013
Female	3223	1103 (34.2)	1.04 (1.00~1.08)	
Ethnicity				
White	4715	1019 (36.5)	1.10 (1.06~1.14)	0.969
Other	2789	1512 (32.1)	1.03 (1~1.07)	
Hypertension				
No	3056	1012 (33.1)	1.08 (1.04~1.12)	0.476
Yes	4448	1519 (34.2)	1.04 (1.01~1.08)	
Congestive heart failure				
No	5156	1649 (32)	1.06 (1.03~1.1)	0.006
Yes	2348	882 (37.6)	1.03 (0.99~1.08)	
Chronic pulmonary disease				
No	5589	1843 (33)	1.07 (1.04~1.1)	0.213
Yes	1915	688 (35.9)	1.03 (0.97~1.08)	
Diabetes				
No	5325	1799 (33.8)	1.07 (1.04~1.1)	0.741
Yes	2179	732 (33.6)	1.02 (0.97~1.08)	
Liver disease				
No	5676	1783 (31.4)	1.06 (1.03~1.1)	0.312
Yes	1828	748 (40.9)	1.04 (1.00~1.09)	
Renal failure				
No	5780	1873 (32.4)	1.07 (1.04~1.1)	0.038
Yes	1724	658 (38.2)	1.03 (0.97~1.09)	
Malignant cancer				
No	6257	1892 (30.2)	1.07 (1.03~1.10)	0.018
Yes	1247	639 (51.2)	1.04 (0.99~1.09)	
Septic shock				
No	4178	1193 (28.6)	1.01 (0.97~1.05)	0.035
Yes	3326	1338 (40.2)	1.09 (1.05~1.12)	
Mechanical ventilation				
No	3763	1168 (31)	0.99 (0.95~1.03)	0.407
Yes	3741	1363 (36.4)	1.09 (1.05~1.12)	
Renal replacement treatment				
No	7142	2363 (33.1)	1.06 (1.03~1.08)	0.318
Yes	362	168 (46.4)	1.04 (0.95~1.14)	
SAPSII				
<40	3530	645 (18.3)	1.03 (0.98~1.09)	0.053
>=40	3974	1886 (47.5)	1.06 (1.03~1.09)	
SOFA				
<8	4076	981 (24.1)	1.04 (0.99~1.08)	0.356
>=8	3428	1550 (45.2)	1.06 (1.03~1.09)	

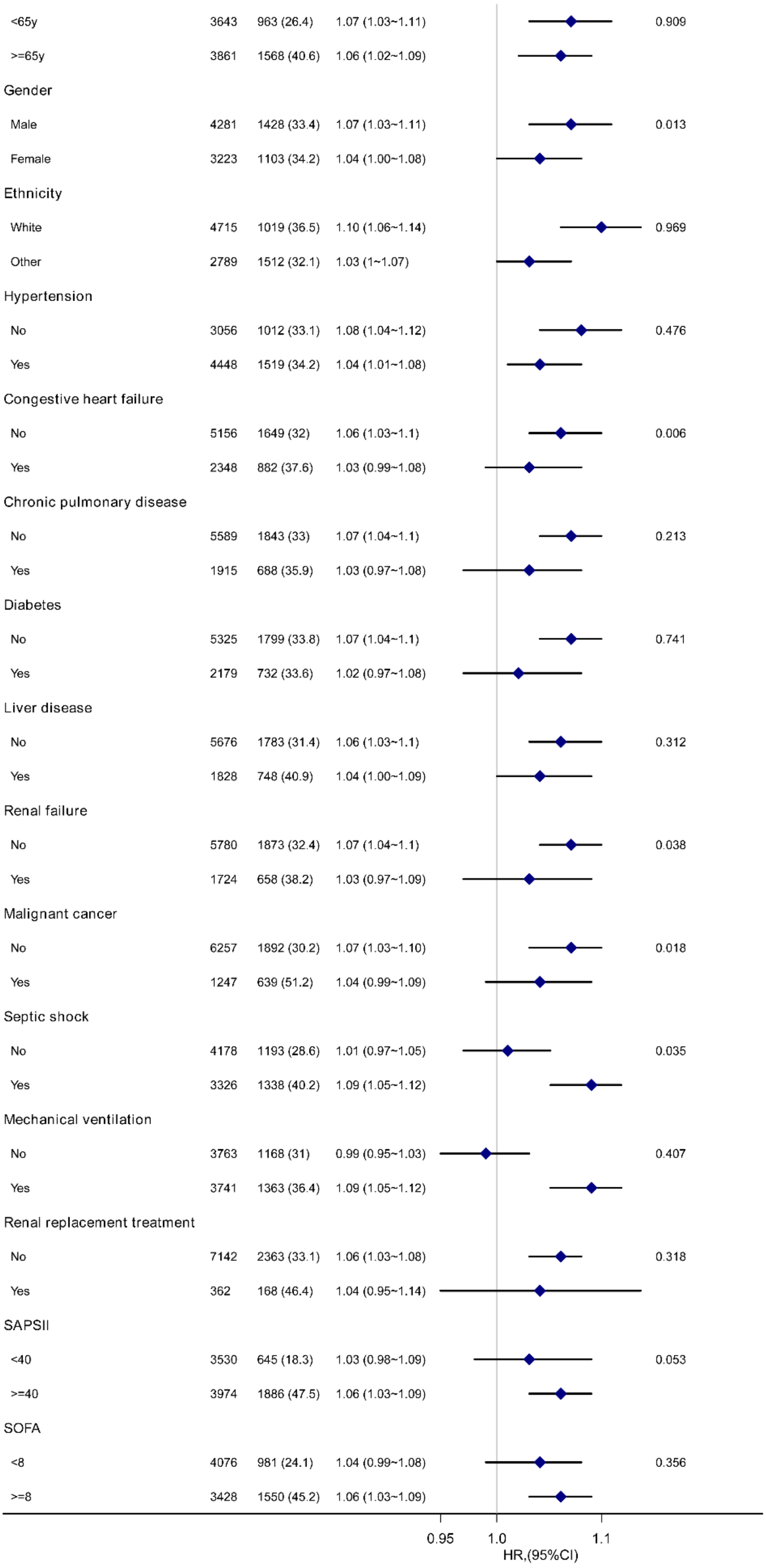

Supplement: Supplementary file 1 — Supplementary figures and table. [file ijmsv20p1165s1.pdf]
